# An evaluation of an innovative screening program based on risk criteria for early diagnosis of head and neck cancers

**DOI:** 10.3389/fpubh.2022.1004039

**Published:** 2023-01-09

**Authors:** Aitor Zabala, Francisco Javier Martín-Arregui, Jon Sagazola, Francisco Javier Santaolalla, Francisco Santaolalla

**Affiliations:** ^1^Otorhinolaryngology Department, Basurto University Hospital, OSI Bilbao-Basurto, IIS BioBizkaia, Basque Health Service/Osakidetza, Bilbao, Spain; ^2^Faculty of Medicine, University of the Basque Country, Bilbao, Spain

**Keywords:** head and neck, screening, risk factor, tobacco, carcinoma

## Abstract

**Introduction:**

Head and neck cancer represents 3% of all cancers and is the cause of 5% of the deaths caused by cancer. The purpose of this study is to evaluate the implementation of a screening program to diagnose the early phase of the head and neck oncological processes.

**Methods:**

We have studied 324 asymptomatic patients who had at least one major risk factor (habitual consumption of tobacco or alcohol) or two minor risk factors: family history of head and neck cancer of the upper aerodigestive tract, occupational exposure, poor oral hygiene and history of Human Papillomavirus or chronic inflammatory processes of the aerodigestive tract. Family and personal head and neck oncological medical history, ENT exploration, performance of CT scans or biopsies and program procedures were analyzed.

**Results:**

The most usual referral criteria for being sent to a specialist was being a smoker (98.1%). 10.5% reported family histories of head and neck cancer, 9.9% reported occupational exposure, 7.1% were referred due to poor oral hygiene and 5.9% were referred for gastroesophageal reflux disease. Although being asymptomatic was a requirement for inclusion, we verified that, after the anamnesis, 9.6% of the patients had some symptom to which they did not give importance to 119 patients (36.7%) presented a lesion that potentially could become malignant, located in the larynx and hypopharynx (25%) and in the oral cavity and oropharynx (10.8%). Eighteen patients (5.56%) presented more than one lesion. The detection rate of neoplasia was 1.2% and the detection rate of pre-neoplastic lesions was 4.6%. There did exist a statistically significant ratio between the detection of pre-neoplastic lesions and occupational exposure to carcinogenic agents (*p* = 0.006), poor oral hygiene (*p* = 0.01) and gastroesophageal reflux disease (*p* = 0.007). Samples were taken for a pathological anatomy study in 30 patients (9.25%). In order to follow up the patients, 22.8% were controlled at hospital medical consultations, 11.1% were examined at outpatient consultation and 66% were given appointments for follow-up visits.

**Conclusions:**

The use of this screening program could be a tool for the early diagnosis of malignant head and neck tumors and to foster healthy habits for cancer prevention.

## 1. Introduction

Malignant tumors originating in the head and neck regions, basically in the upper aerodigestive tract and salivary glands are considered head and neck cancer (1). Tumors of the skin, central nervous system and those of thyroid origin are excluded, since their etiological agents and behavior are different. Head and neck cancer was the seventh most frequent worldwide in the year 2018 and 890,000 new cases were diagnosed and 450,000 deaths occurred due to it (2). According to Siegel et al. (3), it represents 3% of all cancers and somewhat more than 1.5% of all the deaths due to cancer in the United States of America and according to Ferlay et al. (4), it is the cause of 5% of the deaths caused by cancer. The distribution between men and women is 4:1 and the age of appearance is normally over 50 years (3, 5). The changing incidence of the head and neck tumors according to the geographic location and the anatomical situation of the tumor indicates that the etiology of the head and neck tumors is influenced by environmental factors.

### 1.1. Risk factors of head and neck cancer

Smoking and alcoholism are the most important risk factors for suffering head and neck cancer (6, 7). The combination of exposure to tobacco consumption and alcohol simultaneously has a multiplier effect of its carcinogenic capacity in comparison with the individual risk of each of them (7, 8). Thus, while a severe smoker (two packages/day) without consuming alcohol has a risk relative to 2.5, this risk increases significantly if the patient simultaneously presents the two risk factors (9). Hypovitaminosis, nutritional factors, metabolic disorders, occupational exposure to some substances, gastroesophageal reflux disease, Epstein-Barr virus, HPV and the reduction of the protective effect of saliva due to poor oral hygiene are carcinogenic factors that can strengthen the harmful effect of tobacco and of alcohol (9–11).

### 1.2. Head and neck cancer screening programs

Despite the advances in the diagnosis and treatment of head and neck cancer, the overall survival rate of patients has not had the expected improvement in the last 30 years (12, 13).

In head and neck tumors, the independent prognostic factor with greatest repercussion on overall survival is the stage of the tumor. The majority of the head and neck tumors are diagnosed in advanced stages. This fact leads to an increasing morbidity and mortality and, consequently, to healthcare costs (14). On the other hand, the anatomy of the head and neck is especially complex and tumor lesions can appear in areas that do not cause very evident symptoms and that are also difficult to examine. In addition, these carcinomas can appear in more than one location, synchronously or metachronously, in the upper aerodigestive tracts (15) and require specific examinations done by specialists in head and neck cancer for their initial diagnosis (1). Since the treatment of head and neck cancers in their initial stages obtains better results than when they are treated in more advanced stages (14, 16, 17), the setting up of an early detection program of head and neck cancer could be very useful.

Therefore, an early diagnosis program of head and neck cancer as a potential good tool for the clinical manage of this disease has been proposed as working hypothesis. The implementation of a screening program to early diagnose head and neck oncological processes could be reasonably considered to reduce the morbidity and mortality of these oncological processes, improve patient's prognosis and reduce healthcare costs originated by those processes.

## 2. Material and methods

Three hundred twenty four patients, 129 women (39.8%) and 195 men (60.2%), with ages between 50 and 84 years, *m* = 59.38, SD = 7.74 were studied. Women's mean age was 58.29 years, ranging from 50 to 77 years, SD = 6.217 and men's mean age 59.99 years, ranging from 50 to 84 years, SD = 7.29.

The patients were sent to the program from the primary healthcare centers of the Comprehensive Healthcare Organisation to the Otorhinolaryngology Service of the Basurto University Hospital (HUB)/OSI-BilbaoBasurto of the Basque Health Service/Osakidetza during the years 2017 to 2019.

They were asymptomatic patients, over 50 years of age and who had least one major risk factor or two minor risk factors. The habitual consumption of tobacco (>10 cigarettes/day during more than 10 years) and the habitual consumption of alcohol (understood as regular daily consumption or on weekends) were the major risk factors. The five minor criteria were the existence of family history of head and neck cancer of the upper aerodigestive tract, occupational exposure (asbestos, nickel, wood, paint, leather, wool, stone dust, marble, chemical products, coal), poor oral hygiene, history of Human Papillomavirus (HPV) and history of chronic inflammatory processes of the aerodigestive tract or gastroesophageal reflux disease. The following symptoms were considered as exclusion criteria: having suffered a head and neck tumor and the existence of signs or symptoms of possible tumor etiology: dysphonia, dyspnoea, dysphagia, odynophagia, hemoptysis or hematemesis.

An inspection of the head and neck, cervical and facial palpation, exploration of the oral cavity and oropharynx, examination of nasal cavities, rhinopharynx, hypopharynx and pharynx was performed on all the patients, by means of the Olympus ENF-GP2 flexible nasal fiberscope, complementing the examination by means of the Karl Storz 870 CKA rigid 70° laryngeal telescope, with stroboscopic techniques, Olympus CLV-51, or Olympus CV-170 Narrow Band Imaging (NBI).

**Figure 1:**
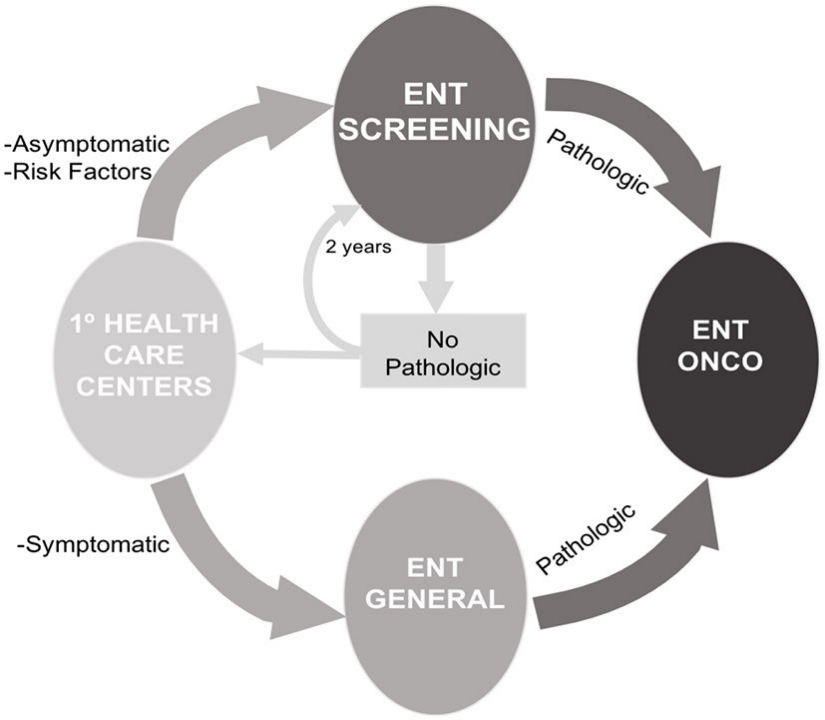
Screening program flowchart.

The patient was sent with a report to the offices of the ORL Service of the HUB in order to carry out the diagnostic and therapeutic tests pursuant to the usual protocols in case positive findings related to head and neck cancer were observed. In the absence of pathological findings, they were given an appointment in a period of 2 years to be re-examined within the Head and Neck Cancer Screening Program. During this period, the patients remained under control of the Primary Care Physician (PCP) to whom they are sent with a report of the findings and the result of the care provided in the program (Figure 1). A document of recommendations in relation to tobacco use and alcohol consumption and the rest of criteria considered risky in head and neck cancer was delivered and explained to the patients.

The analyzed variables were age, gender, tobacco use (moderate <20 cigarettes/day or severe ≥20 cigarettes/day), alcohol consumption, professional activity and contact with carcinogenic products, family or personal history of head and neck cancer, oral hygiene, gastroesophageal reflux disease, detection of neoplastic or pre-neoplastic lesion and its location, request for CT scans and performance of CT scans and performance of biopsy.

### 2.1. Ethical considerations

The research was performed in accordance with principles stated in the Helsinki Declaration. Permission to perform the study in the institution was obtained from the clinical ethic committee of Basurto University (dated September 20, 2017). Additionally, all individuals participating in the research had the aims and procedure explained and provided verbal and written informed consent.

### 2.2. Statistical analysis

Statistical Package for the Social Sciences program, version 17 (SPSS.V17) was employed. We have used the Power Analysis procedures added to SPSS to determinate the minimum sample size needed to detect a hypothesized difference or relationship. The maximum error obtained by this procedure during power estimation was 0.001. Student's *T* test was conducted for the study of independent samples, age in relation to gender, pre-neoplastic findings and the analysis of variance, ANOVA test for the comparison of age and neoplastic findings related to cancer and Levene's independent sample test for equality of variances.

## 3. Results

**Figure 2:**
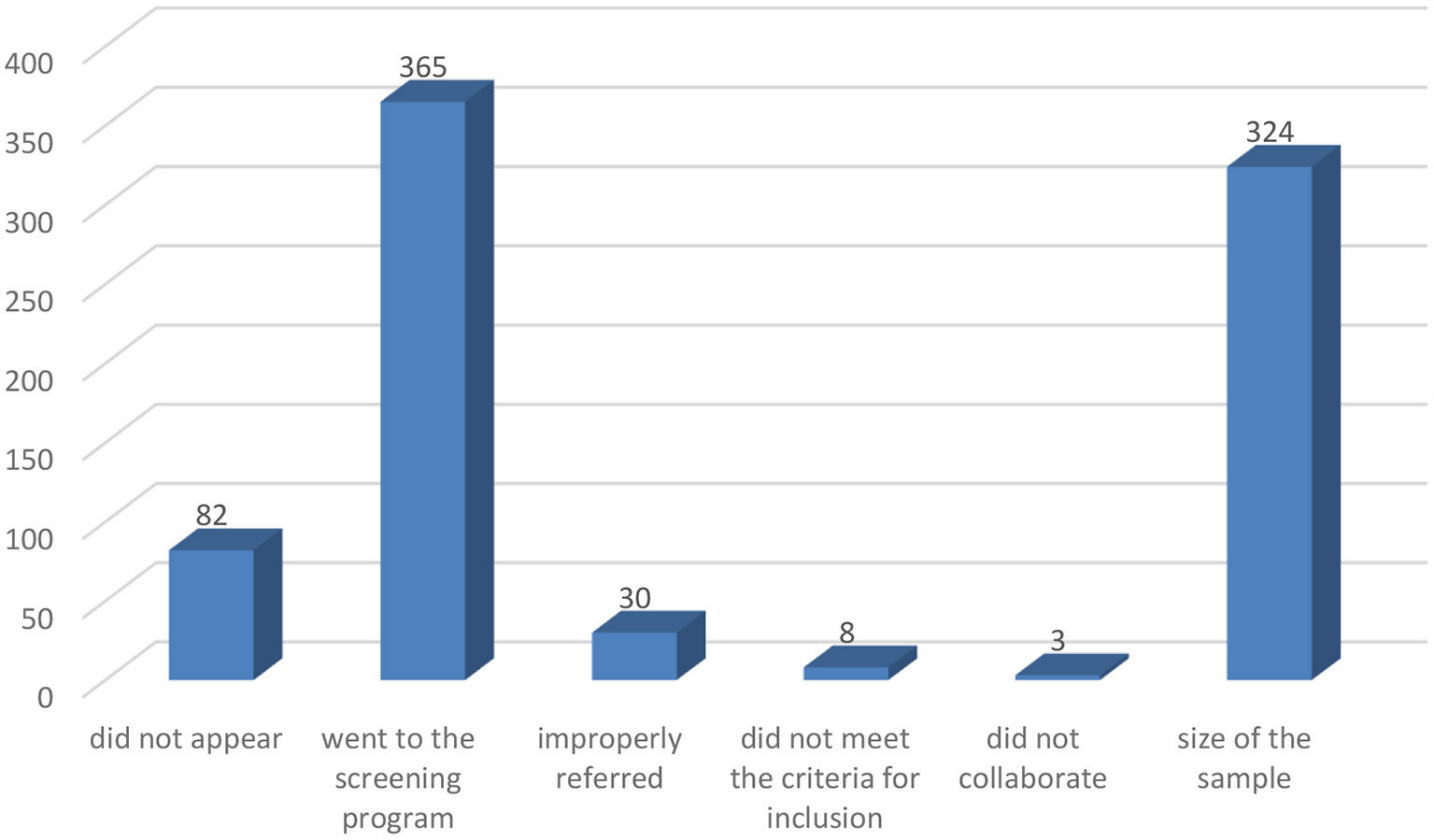
Distribution of the subjects enrolled for screening program.

Of the 447 patients with appointments in the specific medical practice related to the program, 82 did not appear (18.34%). Thirty of the 365 (8.22%) that went to the screening program were improperly referred due to administrative errors and 8 (2.19%) did not meet the criteria for inclusion, so the error rate of appointments making was 10.41%. Three patients that did follow the criteria (0.82%) did not collaborate in the examination. As a result of this, they were not included in the study, hence the final size of the sample was 324 (Figure 2).

### 3.1. Referral criteria

The most usual referral criteria for being sent to a specialist were being a smoker (98.1%), being habitual consumer of alcohol (69.4%), having family histories of head and neck cancer (10.5%), exposure to carcinogenic agents (9.9%), and poor oral hygiene (7.1%).

Thus, 227 patients were referred with 1 Major Criterion, and 95 with both. We found 4 men who habitually consumed alcohol and were not smokers and only 2 cases of women who were neither smokers nor habitual consumers of alcohol.

There are no statistically significant ratios (*p* = 0.743) between the patients' gender and the consumption of tobacco, while there did exist a statistically significant ratio between being male and the consumption of alcohol (*p* = 0.001), occupational exposure to carcinogenic agents (*p* = 0.001), and poor oral hygiene (*p* = 0.006).

The rate of consumption of tobacco and alcohol simultaneously was 29.3% and there was a statistically significant ratio (*p* = 0.007) between the severe habits of tobacco and alcohol consumption, in such a way that 64.7% of the consumers of alcohol considered severe were also severe in the tobacco habit (Table 1).

**Table 1:** Statistical correlations between the detection of lesions and the information collected in the anamnesis or in the examination.

	**No pre-neoplastic lesions**	**Pre-neoplastic lesions**	**1st localization**	**2nd localization**	**Neoplatsic lesions**
Sex	0.686	0.745	**–**	**–**	0.229
Age	**–**	0.377	**–**	**–**	0.982
Tobacco	0.805	0.496	0.864	0.213	0.950
Alcohol	0.529	0.873	0.816	**0.011**	0.146
Tobacco + alcohol	0.321	0.962	**–**	**–**	0.124
Family history head and neck cancer	0.173	0.415	**–**	**–**	0.589
ENT personal history	0.027	**–**	**–**		0.482
Occupational exposure	0.625	**0.060**	**–**	**–**	0.133
Oral hygiene	0.434	**0.010**	**–**	**–**	0.734
Gastroesophageal reflux disease	0.007	**0.007**	**–**	**–**	0.668
Computerized tomography	0.564	**–**	**–**	**–**	**–**
Pathological anatomy	0.404	**–**	**–**	**–**	**–**

Bold values = statistically significant.

Thirty-four patients (10.5%) reported family histories of head and neck and upper aerodigestive tract cancer rising to 41 patients (12.7%) once the anamnesis was done. Thirty-two patients (9.9%) reported occupational exposure; although, once the anamnesis was done, only 28 cases (8.6%) were considered to have authentic exposure to carcinogenic agents. Twenty-three patients (7.1%) were referred due to poor oral hygiene, a figure that increased to 40 (12.3%) after the examination conducted in the visit to the doctor. Nineteen patients (5.9%) were referred for gastroesophageal reflux disease, although the existence of this history was verified in 37 patients (11.4%). That is to say, the minor criteria for referral related to the existence of family oncological histories, poor oral hygiene and gastroesophageal reflux disease were undervalued by the primary care physician as a reason for referring the patients.

Furthermore, although being asymptomatic was a requirement for inclusion, we verified that, after the anamnesis, 31 patients (9.6%) had some symptoms as tiredness, fatigue or occasional fever to which they did not give importance. Likewise, 13 patients (4%) reported personal oncological histories related to head and neck tumors and 86 patients (26.5%) reported having suffered on some occasion symptoms related to the existence of oncological head and neck processes.

### 3.2. Oncological findings

A total of 119 patients (36.7%) presented a lesion that potentially could become malignant, located with preferential incidence in the larynx and hypopharynx (25%) and in the oral cavity and oropharynx (10.8%) with statistically significant distributions. Eighteen patients (5.56%) presented more than one lesion.

Four cases of neoplasia were detected (1.2%) and 15 pre-neoplastic lesions (4.6%). The neoplasms corresponded to four male patients who presented squamous carcinoma with sarcomatoid features of pyriform sinus, one carcinoma of microinfiltrating squamous cells of the vocal cord, one carcinoma *in situ* of the vocal cord and one poorly differentiated squamous carcinoma of the cavum. The pre-neoplastic lesions, 8 men and 7 women, were the following: 6 squamous papillomas and 1 slight/moderate dysplasia of the oral cavity and oropharynx, 6 epithelial hyperplasia's with hyperkeratosis and areas of epidermisation and 1 slight/moderate dysplasia of the larynx and hypopharynx, and 1 inverted papilloma of the nasal cavities.

We did not find the existence of a significant ratio between the age and gender of the patients and the detection of findings of pre-neoplastic and neoplastic lesions. Although all the patients who presented neoplasms (1.2%) or pre-neoplastic lesions (4.6%) were smokers, this correlation was not statistically significant (*p* = 0.827), neither was it related to the severity of tobacco consumption. There were no statistically significant ratios between the detection of neoplasms or pre-neoplasms and the consumption of alcohol or the existence of family or personal histories of having cancer.

There did exist a statistically significant ratio between the detection of pre-neoplastic lesions and the information collected in the anamnesis or in the examination related to occupational exposure to carcinogenic agents (*p* = 0.006), poor oral hygiene (*p* = 0.01) and gastroesophageal reflux disease (*p* = 0.007). Despite the fact that four patients diagnosed with neoplasia were asymptomatic, we verified that, after conducting the anamnesis, there did exist a statistically significant ratio between the presence of symptoms and the detection of pre-neoplastic lesions localized in the larynx and hypopharynx (*p* = 0.001).

The most requested test for the study and staging of the lesions was computerized tomography, on 22 occasions (6.5%). Samples were taken for a pathological anatomy study in 30 patients (9.25%), and 2 samples of different lesions were taken from four of the patients.

As for monitoring the patients, 77 (22.8%) were sent for control at the hospital visits, 36 (11.1%) to the outpatient visits and 66% were given appointments for follow-up visits of the program in a period of 2 years.

## 4. Discussion

In order to optimize resources and improve the cost/effectivity ratio in cancer, it was proposed to focus the early detection programs on the study of target populations that, due to presenting risk factors, were prone to developing the disease. Therefore, screenings were carried out for melanoma, colorectal cancer in patients with inflammatory intestinal disease, retinopathy in premature infants or metabolopathies in neonates. Already in 2005 there were a program directed to the diagnosis of oral cavity tumors in India, where a visual screening in the population that smokes or consumes alcohol has the capacity of preventing deaths secondary to oral carcinomas (18). Nowadays there are good different community survey programs available in India (19–21).

### 4.1. Diagnosis of malignant lesions

In our series, 15 patients (4.6%) were diagnosed with lesions related to cancer and 4 (1.2%) with neoplasia. Shuman et al. (22) describe that 5% of the patients had lesions considered suspicious of neoplasia. After performing biopsies, 7 patients, 0.9% of the total, were diagnosed with malignant/pre-malignant lesions; 2 leucoplakias with dysplasia, 1 papillary carcinoma of the thyroids and 4 epidermoid carcinomas, 3 of them of the larynx and 1 of the oropharynx. The diagnostic rates of malignant/premalignant lesions described by Shuman et al. (23) are slightly below those we obtained, although these differences could be due to the fact that we studied a population with risk factors while Shuman carried out population screening.

In a study of population, community and hospital screenings, proposed by Harris et al. (24), 94 patients (6.81%) of the community screening and 19 (9.04%) of the hospital screening were diagnosed with lesions suspicious of being malignant. In another population program by Freiser et al. (25) of 187 patients, 71 (37.9%) were given appointments for evolutive controls due to which pathological studies were presented, of which 30 patients (16.6% of the total) went to the second assessment. Two patients (1% of the total) were diagnosed with a neoplastic process: 1 cutaneous epidermoid carcinoma and 1 stage IV epidermoid carcinoma of the tonsil. As in the previous studies, by dealing with population screenings, the diagnostic rates are lower. It can be emphasized that the loss of patients between controls is significant, >50%.

In a population screening carried out on the street at NASCAR events, considering the spectators at the event as an at-risk population, 163 patients (43%) with anomalous examinations were referred to specialized centers to complete the study. The majority of patients with pathological findings were men and they smoked more than one package of cigarettes per day. The authors deduced that the individual risk of developing a head and neck carcinoma was increased 1.95 times for each package of cigarettes/day. In the study, the findings of the pathological examinations were not specified and no subsequent follow-up was done on the referral so we do not have premalignant/malignant diagnostic rates (26).

Gourin et al. (27) set some referral criteria according to the findings obtained. Routine controls by their referring physicians were recommended for patients with normal examinations (63%). The patients with normal examination, but with symptoms related to head and neck tumors, benign pathology or patients in which the examination could not be completed for different reasons (26%) were referred to centers with specialists to complete the study or continue routine controls. Lastly, the patients who presented lesions suspicious of being malignant (11%) were referred immediately to an otorhinolaryngologist.

### 4.2. Symptoms

In the screening of Shuman et al. (22), 87% of the patients presented at least one symptom at the time of the examination. The most frequent symptoms were dysphonia (59.8%) and dysphagia (21.8%). Two were symptoms or signs that are statistically associated with suspicious findings of neoplasia: oral pain (11.8%) and the appearance of cervical masses (10%). In another study by Shuman et al. (23), 45% of the patients reported at least one symptom. The most frequent symptoms, as in the previous study, were dysphonia (27%) and dysphagia (21%).

In the studies of screened populations by Harris et al. (24), the most frequent symptoms in hospital-based studies were odynophagia (23.81%), dental and/or gingival problems (16.6%), dysphagia (13.3%) and dysphonia (11.9%). In the community-based screening, the symptoms that were most frequently recorded were dental and/or gingival problems (11.8%), odynophagia (9.2%), dysphagia (5.5%) and dysphonia (5%).

Gourin et al. (27) described that 66% presented some symptoms related to head and neck tumors before making the examination. The most frequent symptom was pain (38%), and 35% of the patients did not relate their symptoms with a potential head and neck tumor disease. There exists, therefore, a lack of knowledge in patients with respect to the symptoms presented by head and neck tumors, so they did not relate their symptoms with the possibility of having a tumor in this location. In this sense, we have to mention that red flag signs of head and neck cancer like non-healing ulcer in mouth, lump in neck, changes in voice or difficulty in swallowing are helpful in early diagnosis and detection of head and neck cancers. Therefore, its consideration will create awareness amongst community regarding it and will help in patients coming for screening at an early stage.

### 4.3. Administrative operation

The recruitment of patients was done in different ways depending on the programs. Shuman et al. (22) offered population screening through announcements in the local communication media, Internet and brochures distributed in public transportation and clinics in areas of low socio-economic levels. Persons interested in the program made an appointment, where they filled out a questionnaire with demographic information and were examined by specialists in otorhinolaryngology.

Harris et al. (24) collected data from two screenings that differed in their recruitment and healthcare site. The community-based screening was promoted by announcements in radio, television, written press and in the website of the la “Head and Neck Cancer Alliance” and was carried out at the Indianapolis Speedway during the NASCAR-organized competition, where the interested patients approached the established stand. After filling out the form with demographic information, the patients were examined by means of indirect laryngoscopy. The hospital-based screening was promoted in the written press and in announcements in the hospital. The patients were tended by otorhinolaryngologists in the Indiana University Hospital and in the Wishard Memorial Hospital of Indianapolis. Both screenings were useful: the community-based screening for the promotion of health and awareness, greater number of patients and low rates of diagnosis of malignant lesions and the hospital-based screening for the detection of lesions in early stages, fewer number of patients, but with a higher rate of diagnosis of malignant lesions.

Freiser et al. (25) proposed three methods to recruit patients: community recruitment (visits to disadvantaged neighborhoods, alcoholics anonymous associations and courses for tobacco discontinuation), recruitment in medical centers (distribution of informative brochures), and recruitment through communication media (announcements in more than 70 media and social networks). The authors concluded that community recruitment allowed collecting a higher proportion of patients with risk factors, and that the recruitment in medical centers was that which attracted more patients.

In our study, only 8 patients (2.19%) were referred in error by their general physician because they did not meet the criteria for inclusion. Thirty patients (8.22%) not belonging to the program were given incorrect appointments due to errors of the administrative system (Figure 2). Although this percentage is not high, their causes have to be studied in order to minimize it, given that the visit time reserved for the screening program is reduced and the period for getting an appointment to go to the program could cause excessive delays.

### 4.4. Acceptance of the screening program among the population

The acceptance of the program among the patients was good, since the appointment attendance index was 81.27%. However, this attendance index decreased over time. In this sense, one has to evaluate putting into motion some strategy to increase the percentage of attendance. To do this, making phone calls or sending SMS messages as a reminder of the date of the appointment has been proposed.

Moreover, the awareness of the risk factors and health education that allows identifying the symptoms related to head and neck cancers and the fostering of healthy habits has been a common and constant objective that was fulfilled in our study. Shuman et al. (23) and Freiser et al. (25) reported that all their patients received recommendations on the cessation of toxic habits and identification of symptoms, both verbally and in writing. Harris et al. (24) stated that 77.98% of the patients in the community-based screening and 86.47% in the hospital-based program reported having broadened their knowledge and increased being alert to the head and neck tumors. Gourin et al. (27) noted that 85% of the patients recognized that the screening to which they were subjected had increased their concern and knowledge on the tumors of this type.

We have to mention as a limitation of this research that we were not able to calculate sensitivity, specificity and positive and negative predictive values of the screening program.

## 5. Conclusions

The screening program for the early diagnosis of head and neck cancer allowed a detection rate of neoplasia of 1.2% and a detection rate of pre-neoplastic lesions of 4.6%. Therefore, the use of this screening program could be a tool for the early diagnosis of malignant head and neck tumors and to foster healthy habits for the prevention of cancer.

## Data availability statement

The original contributions presented in the study are included in the article/supplementary material, further inquiries can be directed to the corresponding author.

## Ethics statement

The study was approved by the Ethical Board of Basurto University Hospital and has been conducted in full accordance with ethical principles, including the World Medical Association Declaration of Helsinki. Written informed consent for participation was not required for this study in accordance with the national legislation and the institutional requirements.

## Author contributions

AZ and FS has contributed to conception and design, data acquisition, analysis, interpretation, drafted, and critically revised the manuscript. FM-A contributed to conception, data acquisition, analysis, drafted, and critically revised the manuscript. JS and FJS contributed to data acquisition, interpretation, drafted, and critically revised the manuscript. All authors contributed to the article and approved the submitted version.
